# Heritable and Lineage-Specific Gene Knockdown in Zebrafish Embryo

**DOI:** 10.1371/journal.pone.0006125

**Published:** 2009-07-03

**Authors:** Mei Dong, Yan-Fang Fu, Ting-Ting Du, Chang-Bin Jing, Chun-Tang Fu, Yi Chen, Yi Jin, Min Deng, Ting Xi Liu

**Affiliations:** 1 Laboratory of Development and Diseases and Key Laboratory of Stem Cell Biology and State Key Laboratory for Medical Genomics, Institute of Health Sciences, Shanghai Institutes for Biological Sciences, Chinese Academy of Sciences & Shanghai Jiao Tong University School of Medicine, and Shanghai Institute of Hematology, RuiJin Hospital, Shanghai Jiao Tong University School of Medicine, Shanghai, People's Republic of China; 2 Shanghai Stem Cell Institute, Shanghai Jiao Tong University School of Medicine, Shanghai, People's Republic of China; 3 Model Organism Division, E-Institutes of Shanghai Universities, Shanghai, People's Republic of China; Universität Heidelberg, Germany

## Abstract

**Background:**

Reduced expression of developmentally important genes and tumor suppressors due to haploinsufficiency or epigenetic suppression has been shown to contribute to the pathogenesis of various malignancies. However, methodology that allows spatio-temporally knockdown of gene expression in various model organisms such as zebrafish has not been well established, which largely limits the potential of zebrafish as a vertebrate model of human malignant disorders.

**Principal Finding:**

Here, we report that multiple copies of small hairpin RNA (shRNA) are expressed from a single transcript that mimics the natural microRNA-30e precursor (mir-shRNA). The mir-shRNA, when microinjected into zebrafish embryos, induced an efficient knockdown of two developmentally essential genes *chordin* and *α-catenin* in a dose-controllable fashion. Furthermore, we designed a novel cassette vector to simultaneously express an intronic mir-shRNA and a chimeric red fluorescent protein driven by lineage-specific promoter, which efficiently reduced the expression of a chromosomally integrated reporter gene and an endogenously expressed *gata-1* gene in the developing erythroid progenitors and hemangioblasts, respectively.

**Significance:**

This methodology provides an invaluable tool to knockdown developmental important genes in a tissue-specific manner or to establish animal models, in which the gene dosage is critically important in the pathogenesis of human disorders. The strategy should be also applicable to other model organisms.

## Introduction

Understanding the development and disease-associated molecular and cellular processes in model organisms has largely relied on gene loss-of-function approaches. Homologous recombination-mediated gene knockout has not yet been achieved in zebrafish, due to the difficulty of generating embryonic stem cell line. The generation of zebrafish knockout has instead taken use of TILLING (targeting induced local lesions in genomes) strategy, in which a library of ENU-mutagenized F1 animals are generated and kept either as a cryopreserved sperm or as a living stock, and the DNA of these animals is screened for genetic lesion in specific exons [1]. Recently, heritable targeted gene disruption with designed zinc-finger nucleases has been reported to inactivate zebrafish *golden*, *ntl* and *vascular endothelial growth factor-2 receptor* genes [2], [3]. While these technologies will undoubtedly speed up the dissection of signal transduction pathways/networks during development and evolution, a conditional knockdown system with stably titering down gene dosage in a tissue-specific fashion is unavailable. This latter strategy is critically important toward establishing zebrafish as a vertebrate model of human pathological conditions and diseases [4], because increasing evidence has demonstrated that haploinsufficiency and epigenetic suppression of tumor suppressor genes, other than complete mutational inactivation or permanent removal of genetic material from the host genome, might be a preferred mechanism in promoting cell transformation [5]. For instance, mice carrying hypomorphic *Sfpi1* enhancer allele that reduces *Pu.1* expression to 20% of normal levels develop acute myeloid leukemia (AML), while a 50% or even a 100% loss of *Pu.1* expression only induc accumulation of abnormal myeloid precursors [6]. Recent studies also indicate that haploinsufficiency of *RPS14* and even lower levels of *α-catenin* expression ranging from 10 to 30% of normal contribute to the pathogenesis of hematological malignant disorders [7], [8]. These data suggest that a narrow window of reduced expression of a tumor suppressor is crucial for acute myeloid leukemia and solid tumor development.

RNA interference (RNAi) using either chemically synthesized small interfering RNAs (siRNA) or DNA-based vector systems expressing small hairpin RNAs (shRNA) driven by RNA polymerase (pol) III promoter has been proved to be an efficient method to mediate sequence-specific, post-transcriptional silencing of virtually any gene in various model organisms [9]. The shRNA-mediated knockdown using either pol III or pol II promoter has been utilized to knockdown gene expression in mammalian cells and animals in a regulated fashion [10], [11]. In combination with a natural backbone of the primary miR-30 microRNA (miRNA), higher amounts of synthetic shRNAs can be produced from the pol III promoter than from the simple hairpin design [12]. This miRNA-based shRNA (mir-shRNA) can also be produced by pol II promoter in cultured cells [13], [14], which offers several advantages over the pol III promoter, including simultaneous expression of several miR-shRNAs from a single polycistronic transcript, and regulated or tissue-specific expression [15]. Unfortunately, a heritable and tissue-specific knockdown of gene expression has not yet been developed in animal model organism such as zebrafish, which largely restricts its genetic potential as a vertebrate model of human disorders associated with reduced expression of etiologic genes.

Here, we show that a miRNA-based shRNA (mir-shRNA), when embedded in an intron of β-actin genomic fragment that is in-frame linked to a fluorescent protein-coding reporter gene and placed under the ubiquitous or tissue-specific pol II promoter, is able to efficiently knockdown the expression of chromosomally integrated and endogenous genes in a heritable and tissue- or cell-specific fashion. Cells with reduced expression of targeted genes can be visualized and dynamically traced owing to the expression of the nontoxic actin-tagged fluorescent protein.

## Results

### Efficient Knockdown of Reporter Gene *In Vivo* by Mir-shRNA

**Figure 1:**
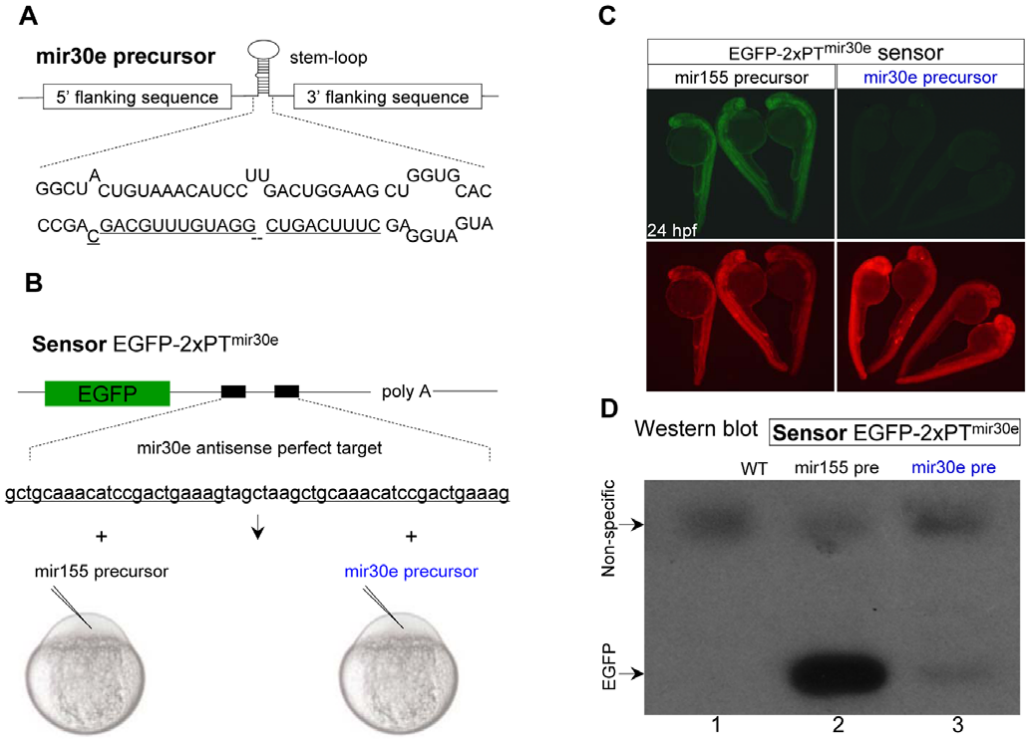
Efficient inhibition of EGFP expression by zebrafish mir-30e precursor *in vivo*. (A, B) Diagram of zebrafish miR-30e precursor sequence (A), and the EGFP sensor EGFP-2×PT^mir30e^ containing two tandem perfectly complementary target sites for miR-30e binding (2×PT) in its 3′UTR (B, upper). The capped EGFP sensor mRNAs were co-injected with either miR-30e or miR-155 precursor mRNAs into one-cell stage embryos (B, bottom). (C) A dramatic decrease of EGFP fluorescence in 24 hpf embryos co-injected with EGFP-2×PT^mir30e^ sensor (10 pg) and miR-30e precursor (50 pg). The DsRed mRNA was co-injected as an injection control (bottom panels). (D) Western blot analysis of 24 hpf embryos injected with EGFP-2×PT^mir30e^ sensor and miR-155 precursor or miR-30e precursor. The upper non-specific bands were shown as a loading control.

It has been previously shown that the 5′ and 3′ flanking sequences of miRNA precursor are crucial for miRNA processing and maturation [16], and the hairpin shRNA can be expressed from a synthetic stem-loop precursor flanked by the 5′ and 3′ flanking sequences of either human miR-30 [14] or mouse miR-155 gene [13]. We first identified zebrafish homologues of mammalian miR-30 and miR-155 genes based on their sequence identity (data not shown), and cloned both zebrafish pri-miR-30e (409 bp) and pri-miR-155 (447 bp) genomic precursor sequences into the pCS2^+^ vector (Figure 1A. and data not shown). Coinjection of *in vitro* synthesized capped pri-miR-30e mRNAs and sensor EGFP mRNAs containing two tandem perfectly complementary target sites (2×PT for miR-30e binding) in its 3′UTR (EGFP-2×PT^mir30e^; Figure 1B) into one-cell stage embryos, resulted in a striking decrease of both EGFP fluorescence and proteins (Figure 1C, D, right panels). As a control, injection of miR-155 did not have any effects on the expression of EGFP-2×PT^mir30e^ (Figure 1B–D, left panels). Compared with the mir-30e, injection of the capped miR-155 mRNAs showed less efficiency to knockdown the EGFP sensor containing 2×PT for miR-155 binding (EGFP-2×PT^mir155^) (data not shown). The result suggests that the 409 bp of pri-miR-30e precursor contains flanking sequence capable of directing the production of functionally mature miR-30e *in vivo*. We therefore used miR-30e precursor as a backbone in the following experiments.

**Figure 2:**
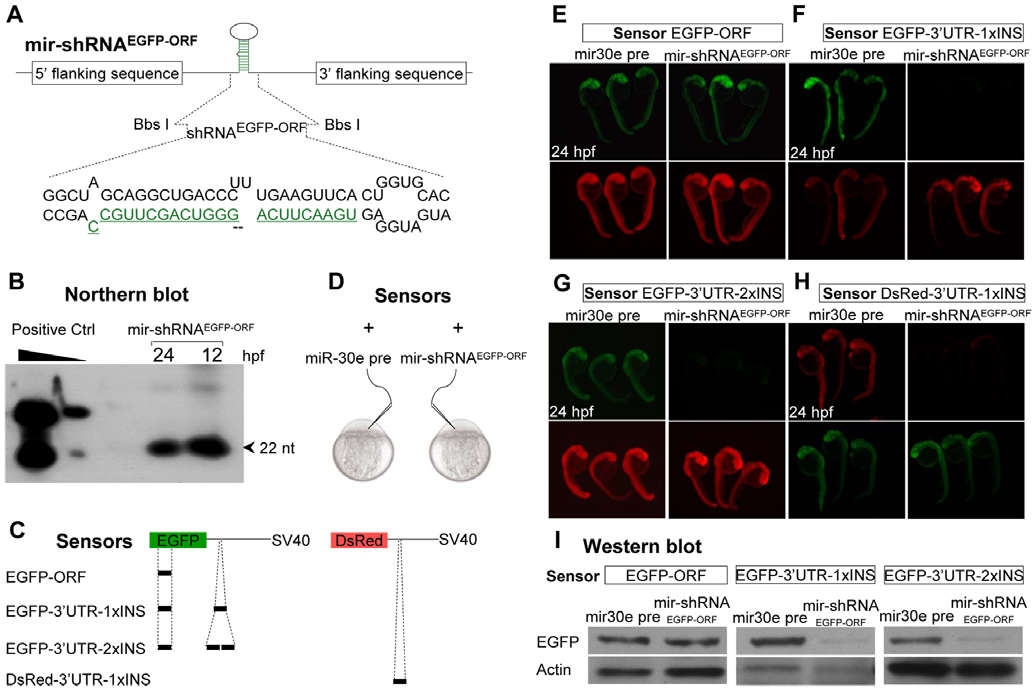
Knockdown of EGFP sensors by mir-shRNA *in vivo*. (A) Diagram of miRNA-based shRNA^EGFP-ORF^ (mir-shRNA^EGFP-ORF^). The stem-loop region of miR-30e precursor was replaced with chemically synthesized shRNA^EGFP-ORF^ oligonucleotides containing the same sequence as the miR-30e stem-loop, except that the miR-30e hairpin stem was changed to the sequence that was complementary to the open reading frame (ORF) of EGFP transcript. (B) Northern blot analysis of 12 and 24 hpf embryos injected with *in vitro* synthesized mir-shRNA^EGFP-ORF^ mRNAs. The U6 promoter-driven expression of shRNA^EGFP-ORF^ in 293T cells was used as a positive control (left lanes). (C) Diagram of various sensors containing one or two copies of binding sequence for mir-shRNA^EGFP-ORF^ within the ORF or the 3′UTR. The 22-bp long binding sequence was inserted into the 3′UTR-SV40 of either EGFP or DsRed gene. (D) Individual capped sensor mRNAs was co-injected with either miR-30e precursor control or mir-shRNA^EGFP-ORF^. (E–H) Detection of EGFP and DsRed fluorescence in 24 hpf embryos injected with various sensor mRNAs. Red fluorescence was used as an injection control in E, F and G, and green fluorescence as injection control in H. (I) Western blot analysis of 24 hpf embryos shown in panels E–G. The β-actin was used as a loading control.

We next replaced the miR-30e stem-loop sequence with a 24 nt-long linker containing two Bbs I restriction sites that allowed insertion of a synthesized shRNA^EGFP-ORF^ stem-loop and preserved all flanking sequences intact (Figure 2A). The resultant construct mir-shRNA^EGFP-ORF^ contained the same sequence (including a di-nucleotide bugle [17]) as the native miR-30e precursor, except that the strand of the mir-30 hairpin stem has been replaced with the 22 nt-long sequences complementary to *EGFP* open reading frame (ORF) at the position of 121–142 (Figure 1A and Figure 2A). Northern blot analysis showed that the mir-shRNA^EGFP-ORF^ mRNAs, when injected into one-cell stage embryo, gave rise to abundant mature shRNA^EGFP-ORF^ fragment in 12 and 24 hours post fertilization (hpf) embryos (Figure 2B). To test whether the mir-shRNA^EGFP-ORF^ was able to knockdown the EGFP expression, we first microinjected the EGFP-ORF sensor (containing the targeted site within its ORF; Figure 2C, top) with either miR-30e precursor control or mir-shRNA^EGFP-ORF^ into one-cell stage embryos (Figure 2D). Surprisingly, no obvious knockdown effect was observed in 24 hpf embryos as evidenced by fluorescence and Western blot analysis (Figure 2E, I, left panels), although the same shRNA^EGFP-ORF^ under H1 promoter was able to efficiently knockdown the EGFP expression in transfected 293T cells (Figure S1).

We next tested additional sensors EGFP-3′UTR-1×INS and EGFP-3′UTR-2×INS, in which one and two copies of the same targeted site were introduced into the SV40-3′UTR of EGFP sensors (Figure 2C, middle). An obvious reduction on both EGFP fluorescence and proteins was observed (Figure 2F, G; Figure 2I, right panels). The same result was also obtained when the targeted site was inserted into the 3′UTR of the DsRed sensor (Figure 2C, bottom; Figure 2H). In contrast, injection of miR-30e precursor control had no detectable effects on EGFP expression (Figure 2E–I, left panels). These results suggest that the targeted sites within the 3′UTR preferably conferred the knockdown effects by mir-shRNAs, consistent with previous observations [18].

To further validate the knockdown effects conferred by 3′UTR, we designed two shRNAs, designated as mir-shRNA^EGFP-SV40-1^ and mir-shRNA^EGFP-SV40-2^, against the proximal and distal sites within the SV40 of EGFP sensor (Figure S2A). Both mir-shRNA^EGFP-SV40-1^ and mir-shRNA^EGFP-SV40-2^, when co-injected with the capped EGFP-SV40 sensor into one-cell stage embryos, were able to induce a dramatic knockdown of EGFP fluorescence and proteins (Figure S2B, C). Furthermore, injection of either mir-shRNA^EGFP-SV40-1^ or mir-shRNA^EGFP-SV40-2^, but not mir-shRNA^EGFP-ORF^ mRNAs, suppressed the EGFP expression in the hematopoietic intermediate cell mass (ICM) and forebrain in 22 hpf embryos derived from a transgenic line Tg(*zgata-1:EGFP-SV40*) that stably expresses EGFP driven by *gata-1* promoter (Figure S2D,E).

### Efficient Knockdown of Endogenous Target Genes by Mir-shRNA

To test whether the mir-shRNA could knockdown endogenous genes in zebrafish, we selected *chordin* and *alpha-catenin* that were expressed during early embryogenesis. It has been shown that loss-of-function of *chordin* results in embryonic ventralization with the expansion of mesodermal hematopoietic tissue at the expanse of neuroectodermal development [19]. Significantly reduced expression of *alpha-catenin* has been observed in the leukemia-initiating cells of del(5q)-associated acute myeloid leukemia/myelodysplastic syndrome and in the invasive solid tumors [8], [20].

**Figure 3:**
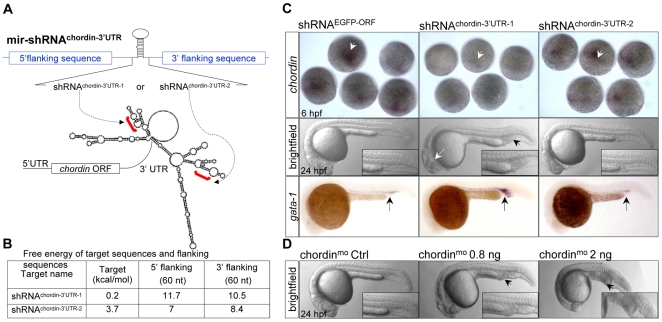
Knockdown of endogenous cellular *chordin* expression. (A) Diagram of mir- shRNA^chordin-3′UTR-1^ and mir-shRNA^chordin-3′UTR-2^ against the 3′UTR of *chordin* gene, whose predicted secondary structure was shown at the bottom. Red brackets denoted the targeted regions. (B) Free-energy of the targeted regions and corresponding flanking sequence predicted with mFold software. (C) Phenotypes of *chordin*-deficient embryos. WISH analysis of *chordin* expression in the 6 hpf embryos injected with 200 pg of shRNA^EGFP-ORF^ (control), shRNA^chordin-3′UTR-1^ or shRNA^chordin-3′UTR-2^ mRNAs using a dig-labeled full-length 3′UTR of *chordin* as probe (upper panels). A significantly enlarged ICM (black arrowhead) with a partial loss of neural tissues (white arrow, 61/99) were observed in 24 hpf embryos injected with only shRNA^chordin-3′UTR-1^, but not with shRNA^chordin-3′UTR-2^ and control (middle panels). A higher level of *gata-1* expression was also detected in the shRNA^chordin-3′UTR-1^-injected embryos, compared to control or shRNA^chordin-3′UTR-2^-injetced embryos (bottom panels). (D) Morphology of embryos injected with *chordin* morpholino oligonucleotides and its 4-base pair mismatch control. Embryos at 6 hpf are dorsal view, and embryos at 24 hpf are lateral views with head to the left.

Because the local secondary structure and the free energy (ΔG) of 3′UTR might affect the accessibility by mir-shRNA [16], we selected two sequences within the 3′UTR of *chordin* gene, which could be potentially targeted by mir-shRNA^chordin-3′UTR-1^ and mir-shRNA^chordin-3′UTR-2^, respectively (Figure 3A). These two sequences were selected with mFold software [21] based on the ΔG of these sites and their flanking sequence (60 bp 5′ and 3′), which the mir-shRNA^chordin-3′UTR-1^ appeared to have lower ΔG than mir-shRNA^chordin-3′UTR-2^ (Figure 3B). The capped mir-shRNA^chordin-3′UTR-1^ and mir-shRNA^chordin-3′UTR-2^ was individually microinjected into one-cell stage embryos and whole-mount in situ hybridization (WISH) analysis with a dig-labeled antisense probe was performed to evaluate the level of *chordin* transcripts. While the *chordin* transcripts were appropriately detected in the dorsal shield of wild-type or mir-shRNA^EGFP-ORF^ control-injected embryos at 6 hpf as previously reported [22] (Figure 3C, left panel, white arrowhead), a dramatic reduction of *chordin* transcripts was observed in the embryos injected with 200 pg of mir-shRNA^chordin-3′UTR-1^, but not with the same amount of mir-shRNA^chordin-3′UTR-2^ likely due to its higher ΔG (3.7 v.s. 0.2 kcal/mol) (Figure 3B and 3C, white arrowheads). As a result, an enlarged blood ICM (Figure 3C, black arrowhead, n = 61/99) with increased *gata-1* expression (black arrow) and partial loss of neural tissues (white arrow) were observed only in mir-shRNA^chordin-3′UTR-1^-injected embryos at 24 hpf, which were comparable to the embryos injected with 0.8 ng of *chordin*-specific morpholino oligonucleotides [19] (Figure 3D).

**Figure 4:**
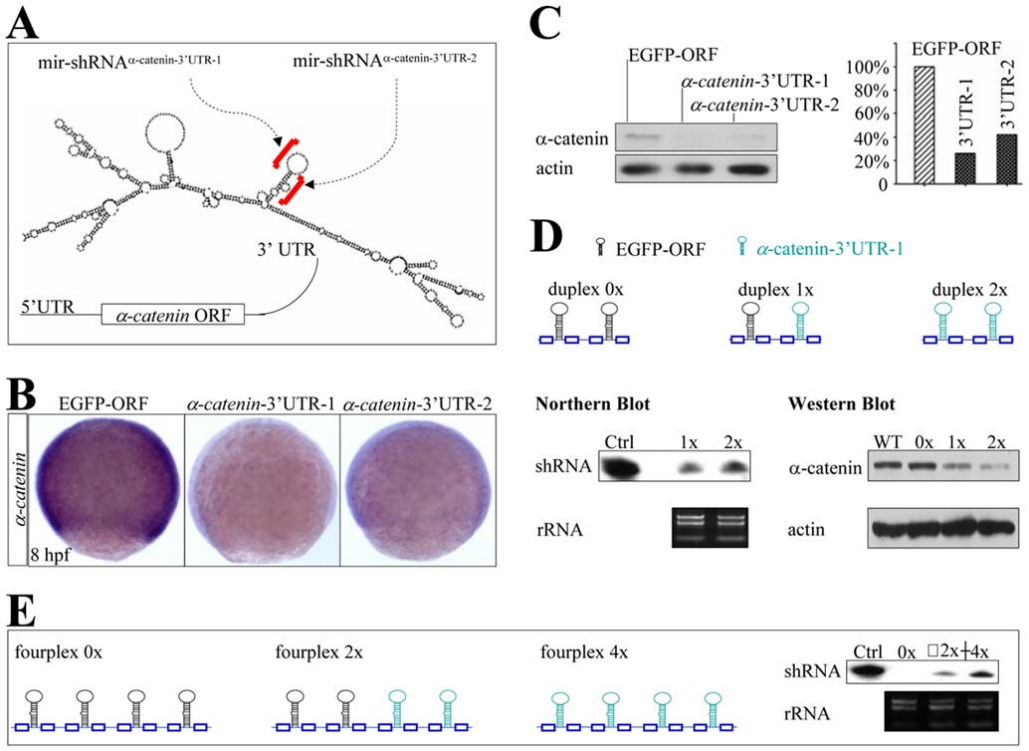
Knockdown of endogenous *α-catenin* expression. (A) Diagram of mir-shRNA^α-catenin-3′UTR-1^ and mir-shRNA^α-catenin-3′UTR-2^ against the 3′UTR of *α-catenin* gene, whose predicted secondary structure was shown at the bottom. Red brackets denoted the targeted regions. (B) WISH analysis of *α-catenin* expression in the 8 hpf embryos injected with 160 pg of either shRNA^EGFP-ORF^, shRNA^α-catenin-3′UTR-1^ or shRNA^α-catenin-3′UTR-2^ mRNAs. (C) Quantitative Western blot analysis of α-catenin protein in 22 hpf embryos injected with shRNAs shown in panels B (5 embryos for each lane). (D) Diagram of mir-shRNA duplexes carrying two copies of shRNA^EGFP-ORF^ (duplex 0×), one copy for each shRNA^EGFP-ORF^ and shRNA^α-catenin-3′UTR-1^ (duplex 1×), and two copies of shRNA^α-catenin-3′UTR-1^ (duplex 2×). Northern and Western blot analyses of 22 hpf embryos injected with shRNAs 0×, 1× or 2× as shown at the bottom (100 embryos for each line). The ribosomal 5S RNA and β-actin was used as a loading control, respectively. (E) Diagram of mir-shRNA fourplexes carrying four copies of shRNA^EGFP-ORF^ (0×), two copies for each shRNA^EGFP-ORF^ and shRNA^α-catenin-3′UTR-1^ (2×), and four copies of shRNA^α-catenin-3′UTR-1^ (4×). Northern blot analysis of 22 hpf embryos injected with shRNAs 0×, 2× or 4× (100 embryos for each line). The ribosomal RNA was used as a loading control.

The mir-shRNA^α-catenin-3′UTR-1^ and mir-shRNA^α-catenin-3′UTR-2^ were also designed to target two regions within the 3′UTR of *alpha-catenin* gene (Figure 4A). WISH analysis showed that the *alpha-catenin* was maternally expressed (data not shown) and ubiquitously detected in wild type or control mir-shRNA^EGFP-ORF^-injected embryos at 8 hpf (Figure 4B, left panel). In contrast, a significant reduction in the *alpha-catenin* transcripts was consistently observed in the 8 hpf embryos injected with 160 pg of either mir-shRNA^α-catenin-3′UTR-1^ or mir-shRNA^α-catenin-3′UTR-2^ (Figure 4B, right panels). Consistently, quantitative Western blot analysis showed that the alpha-catenin proteins were dramatically decreased to 26% of normal level at 22 hpf (Figure 4C). To determine whether the mir-shRNA^α-catenin-3′UTR-1^ can confer gene knockdown in a dosage-dependent fashion, embryos were injected with the same amount of duplex 0×, duplex 1× and duplex 2×, which harbored zero, one and two copies of shRNA^α-catenin-3′UTR-1^, respectively (Figure 4D, top). Northern and Western blot analyses showed that as expected, injection of duplex 2× generated about one-fold more shRNAs (Figure 4D, bottom) and one-fold less α-catenin proteins than injection of duplexes 1× and control at 22 hpf embryos (Figure 4D, bottom). Consistently, injection of fourplex 4× also generated one-fold more shRNAs than injection of fourplex 2× (Figure 4E), suggesting that the α-catenin protein could be further reduced (data not shown). Thus, the experimental design presented here provided not only an efficient means to screen and identify mir-shRNA capable of reducing target gene level, but also a feasible tool to titer down the gene dosage in a controllable manner.

### Intronic Mir-shRNA Expression and Genetic Tractability under Pol II Promoter

**Figure 5:**
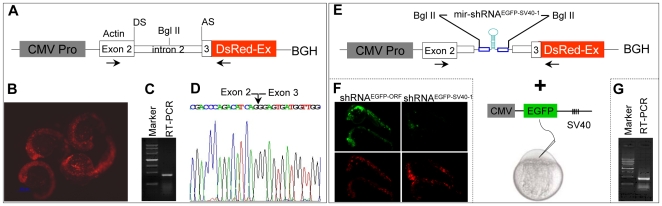
Design of pol II promoter-driven knockdown construct. (A) Diagram of pol II-type promoter CMV driven-knockdown vector (*CMV promoter-*actin-DsRed-BGH). DS: donor site; AS: acceptor site. The first 21-base pairs of exon 3 of zebrafish *actin* gene have been in-frame fused to the DsRed fluorescent protein gene followed by a bovine growth hormone (BGH) sequence as 3′UTR. (B) Red fluorescence was observed in 22 hpf embryos injected with the plasmid shown in panel A. (C) RT-PCR analysis with total RNAs derived from 22 hpf embryos shown in panel B. The primers used are indicated by horizontal arrows in panel A. (D) The sequence of RT-PCR product shown in pane C. Note that the entire intron 2 of the *actin* gene has been spliced out (arrow). (E) The mir-shRNA^EGFP-SV40-1^ was inserted into the intron 2 at the Bgl II site and co-injection with CMV-EGFP-SV40 reporter plasmid. (F) Knockdown of EGFP fluorescence in the 24 hpf embryos co-injected with EGFP-SV40 reporter plasmid plus *CMV promoter-actin-DsRed-BGH* plasmid carrying either mir-shRNA^EGFP-ORF^ or mir-shRNA^EGFP-SV40-1^. (G) RT-PCR analysis of total RNAs derived from 22 hpf embryos shown in panel F.

Natural miRNAs lying within the intron of protein-coding genes have been shown to be co-transcribed with message mRNAs under ubiquitous or tissue-specific pol II promoters [16]. We designed a cytomegalovirus (CMV) prompter-driven expression cassette in which the zebrafish *β-actin* genomic fragment containing an intact exon 2 (123 base pairs), an intact intron 2 (364 base pairs) and the first 21-base pairs of exon 3, was in-frame fused to the DsRed-Express (DsRed-EX) reporter followed by a bovine growth hormone (BGH) poly (A) site as 3′UTR (Figure 5A). After injection of the plasmid cassette into one-cell stage embryos, the injected embryos showed red fluorescence due to the expression of the chimeric β-actin-DsRed protein, and no any morphological and developmental abnormalities were observed during embryogenesis (Figure 5B). The precise splicing of intron 2 from the *β-actin*-*DsRed* fusion gene *in vivo* was confirmed by amplification of a predicted size of RT-PCR product and subsequent sequencing (Figure 5C, D). Furthermore, co-injection of the plasmid expression cassette carrying an introduced mir-shRNA^EGFP-ORF^ or mir-shRNA^EGFP-SV40-1^ at the Bgl II restriction site within the intron 2, with the EGFP-SV40 reporter plasmid (Figure 5E), resulted in a dramatic decrease of EGFP fluorescence with correct splicing of the mir-shRNA-containing intron 2 in 22 hpf embryos (Figure 5F, G). The results provide a proof-of-concept that knockdown of chromosomally integrated or endogenous genes under a tissue- or lineage-specific pol II promoter might be feasible.

### Heritable and Lineage-specific Knockdown of Chromosomally Integrated and Endogenous Genes in the Developing Erythroid Progenitors and Hemangioblasts during Embryogenesis

We previously established a transgenic reporter line Tg(*zgata-1:EGFP-SV40*) with stable expression of EGFP under the erythroid-specific *gata-1* promoter [23]. The transgenic line is unique in that the EGFP expression can be detected in multiple tissues including the midbrain, forebrain, dorsal neurons other than in the erythropoietic ICM, which has also been observed in previous transgenic line with the same *gata-1* promoter [24]. Thus, this line offers a unique advantage as a reporter line to detect mir-shRNA mediated knockdown effects in multiple lineages and tissues within an individual animal.

**Figure 6:**
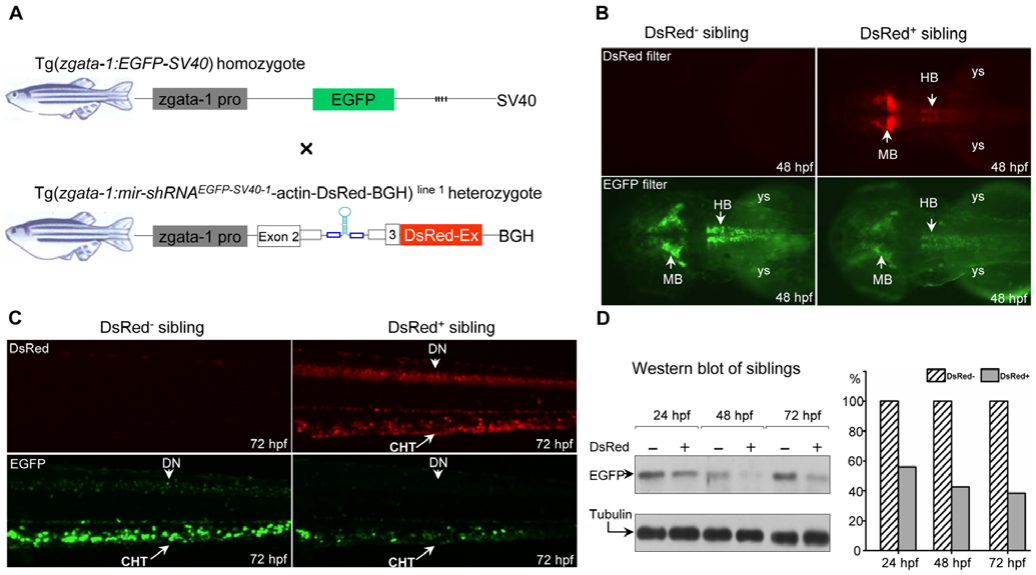
Tissue-specific knockdown of chromosomally integrated EGFP expression. (A) Diagram of transgenic lines Tg(*zgata-1:EGFP-SV40*) and Tg(*zgata-1:mir-shRNA^EGFP-SV40-1^-*actin-DsRed-BGH) ^line 1^ under control of zebrafish *gata-1* promoter. (B) Knockdown of EGFP fluorescence was observed in the mid- and hindbrain of the DsRed^+^, but not DsRed^−^ F2 sibling at 48 hpf. Embryos are dorsal view with head to the left. (C) Knockdown of EGFP fluorescence was observed in the dorsal neurons and caudal hematopoietic tissue of the DsRed^+^, but not DsRed^−^ F2 sibling at 72 hpf. MB: midbrain; HB: hindbrain; ys: yolk sac; DN: dorsal neurons; CHT: caudal hematopoietic tissue. Embryos are lateral view with head to the left. (D) Western blot analysis of EGFP expression in the DsRed^−^ and DsRed^+^ F2 embryos at 24, 48 and 72 hpf. The α-tubulin protein was used as a loading control.

We screened and established 6 transgenic lines stably expressing the intronic mir-shRNA^EGFP-SV40-1^ under the same *gata-1* promoter. One of the lines designated as Tg(*zgata-1:mir-shRNA^EGFP-SV40-1^*-actin-DsRed-BGH)^line 1^ was selected to determine its knockdown potency because the DsRed fluorescent proteins were also observed to be expressed in the same tissues as the reporter line Tg(*zgata-1:EGFP-SV40*). When the homozygous Tg(*zgata-1:EGFP-SV40*) reporter line was crossed to heterozygous Tg(*zgata-1:mir-shRNA^EGFP-SV40-1^*-actin-DsRed-BGH)^line 1^ (Figure 6A), 421 (52.9%) and 375 (47.1%) of 796 F2 embryos collected from multiple crosses were DsRed^+^ and DsRed^−^, respectively, suggesting a dominant Mendelian ratio. The EGFP expression in both mRNA and protein levels was dynamically evaluated in the DsRed^+^ and DsRed^−^ sibling embryos at 24, 48 and 72 hpf. The results demonstrated a significant reduction of EGFP fluorescence and transcripts in the midbrain (MB), hindbrain (HB), dorsal neurons (DN) and caudal hematopoietic tissue (CHT) only in the DsRed^+^ embryos at 48 and 72 hpf, compared with the appropriate expression of EGFP in the DsRed^−^ siblings at the same developmental stages (Figure 6B, C, arrows; Figure S3). Western blot analysis further confirmed the results that a 45%, 58% and 62% of total EGFP protein was lost in the DsRed^+^ embryos at 24, 48 and 72 hpf, respectively (Figure 6D). The results indicate that the *mir-shRNA^EGFP-SV40-1^* is able to mediate the cell subtype-specific knockdown of a chromosomally integrated gene in a genetically heritable manner.

**Figure 7:**
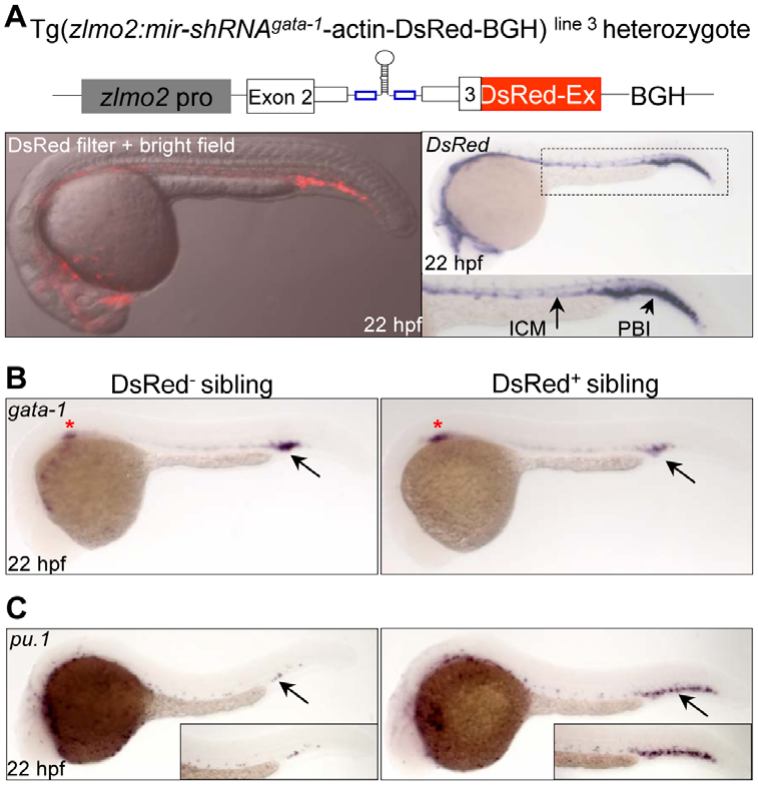
Tissue-specific knockdown of endogenous *gata-1* expression. (A) The expression of DsRed fluorescence and transcripts recapitulates the endogenous *lmo2* expression pattern in the Tg(*zlmo2:mir-shRNA^gata-1^*-actin-DsRed-BGH) ^line 3^. Note that stronger expression of *DsRed* transcripts can be observed in the PBI region than in the ICM region. ICM: intermediate cell mass; PBI: posterior blood island. (B) WISH analysis of *gata-1* expression in the DsRed^−^ and DsRed^+^ F1 siblings at 22 hpf. Red star denotes the *gata-1* staining in the developing kidney. (C) WISH analysis of *pu.1* expression in the DsRed^−^ and DsRed^+^ F1 siblings at 22 hpf. Note that the pu.1 staining is massively increased (arrow). All embryos are dorsal view with head to the left.

To further test the knockdown effects on endogenous genes, we selected an erythroid-specific *gata-1* gene to evaluate the knockdown the erythroid-specific *gata-1* gene in the developing hemangioblasts. A transgenic line Tg(*zlmo2:mir-shRNA^gata-1^*-actin-DsRed-BGH)^line 3^ stably expressing the *mir-shRNA^gata-1^* under the hemangioblastic *lmo2* promoter [25], [26] was established (Figure 7A). Fluorescence and WISH analyses showed that the DsRed fluorescence and transcripts were specifically expressed in the *lmo2*-positive vascular endothelial cells and hematopoietic progenitors at the ICM and posterior blood island (PBI) as observed previously [25] (Figure 7A). As expected, a 50% reduction of *gata-1* transcripts was only observed in the PBI of all DsRed^+^ siblings at 22 hpf (Figure 7B, arrows). More importantly, the *pu.1* transcripts (a myeloid progenitor-specific gene) were concomitantly increased in the same region of all DsRed^+^ sibling observed at 22 hpf (Figure 7C, arrows). The result is consistent with previous observations that reciprocal negative regulation between pu.1 and gata1 determines myeloid versus erythroid fate [27].

## Discussion

In this study, we have developed a novel methodology that uses a microRNA-based shRNA (mir-shRNA) to reduce the dosage of a given gene in a controllable and tissue-specific manner. Although the miRNA-based shRNA knockdown strategy has been successfully used to mediate efficient and specific knockdown of genes *in vitro*, its use in combination with cell- or tissue-specific pol II promoter in animals is still absent. The backbone of miR-30 is one of the most frequently used microRNA sequence to direct the processing and maturation of shRNA, because its stem sequence could be substituted with exogenous sequences that match different target genes and to produce 12 times more mature shRNAs than simple hairpin designs [12], [14], and its ability to prevent interferon-stimulated gene expression and associated off-target effects and toxicity in cultured cells and mouse brain [17], [28].

Although the sequences targeted by mir-shRNA in this study are derived from 3′UTR, the mir-shRNA should be able to target sequences within other part of a given transcript such as the open reading frame as described previously [12], [17]. The observations that the targeted site in the 3′UTR, rather than in the ORF of EGFP, confer robust knockdown effects by mir-shRNA^EGFP-ORF^ (Figure 2), suggest that the mir-shRNA might preferably target sequence in the 3′UTR *in vivo*. Interestingly, the similar phenomenon has also been observed in cultured Schneider S2 cells, although the underlying mechanism remains elusive [18]. On the other hand, to optimize the site that mediate maximal knockdown effects, two to three potential target sequences for a given gene should be designed with mFold software and selected based on the predicted secondary structure and ΔG. Furthermore, as shown in Figure 4D and 4E, taking use of mir-shRNA duplex or fourplex also provides a potential means to maximally knockdown the target genes whose dosage can be regulated in a controllable fashion.

The use of pol II promoter-driven mir-shRNA expression cassette provides a unique advantage in that the cells or tissues with reduced expression of target gene can be genetically traced and visualized in the transparent zebrafish embryos, because of the simultaneous expression of the chimeric red fluorescent protein, β-actin-DsRed. Transgenic embryos and adults stably expressing this chimeric fluorescent protein appear morphologically and developmentally normal and have been fertile for three generations, suggesting a lack of detectable toxicity. Given the facts that reduced expression of many disease-associated and developmentally important genes due to either epigenetic inactivation or haploinsufficiency, contribute to the pathogenesis of myeloid malignancies and tumorigenesis [6], [8], the methodology described in this study highly complements the recently reported zinc finger-mediated gene knockout strategy in zebrafish, and provide an invaluable tool to knockdown disease-associated gene in specific tissues or cells in the model organisms. In combination with Cre-loxP recombination and drug-inducible strategy [25], [29], the pol II promoter-driven mir-shRNA knockdown system could be further optimized to prevent embryonic lethality from reduced expression of the developmentally crucial genes and tumor suppressors.

## Materials and Methods

### Fish care

The maintenance, breeding and staging of zebrafish lines (Tubingen and Shanghai) were performed as described previously [30].

### Cloning and plasmid construction

The precursor sequences of zebrafish mir30e (409 bp) and mir155 (447 bp) were cloned from the genomic DNA of Tubingen adult fish into the pCS2^+^ vector. The 68-bp mir30e stem-loop region was replaced with a linker sequence containing two Bbs I sites using two-step PCR. The shRNA sequences were synthesized as DNA oligonucleotides (Invitrogen) and inserted at the Bbs I sites. The sensor sequences (2×PT) were synthesized as DNA oligonucleotides (Invitrogen) and placed into the 3′UTR of the pCS2^+^-EGFP plasmid. The genomic sequence of zebrafish β-actin containing an intact exon 2 and intron 2, and the first 21-bp of exon 3 was in-frame infused to the open reading frame of DsRed-3′UTR BGH, and cloned into the pCS2^+^ plasmid through BamH I and EcoR I sites. The mir-shRNA was inserted into an endogenous Bgl II site within the intron 2. The resultant intronic mir-shRNA was then inserted downstream of the *gata-1* promoter or *lmo2* promoter and cloned into the I-SceI-containing plasmid. All primer sequences were available in Table S1.

### Microinjection and establishment of transgenic zebrafish line

All capped mRNAs were synthesized with SP6 mMessage mMachine (Ambion) and microinjected into one-cell stage embryos. The transgenic plasmids flanked by the I-SceI sites were prepared with endotoxin-free miniprep kit (Axygen). Microinjection was performed at one-cell stage embryos with 2 nl of injection solution containing 40 pg/nl of DNA, 0.5×I-SceI buffers and 0.5 units/µl I-SceI meganuclease (New England Biolabs). Injected embryos were raised to sexual maturity (F0 founders) and crossed to wild-type zebrafish to generate F1 progeny, which were screened for red fluorescent DsRed expression in the ICM at 24 hpf. The DsRed^+^ F1 embryos were raised to adults to establish the stable transgenic lines. Embryos were imaged using a Zeiss SteREO Discovery V12 fluorescent stereomicroscope.

### shRNA Northern blot analysis and RT-PCR

Total RNAs of embryos injected with capped mRNAs were extracted with Trizol (Invitrogen), and separated on 12% of UREA-PAGE gel. Northern blot was probed with dig-labeled antisense probe, and visualized using DIG luminescent detection kit for nucleic acids (Roche). RT-PCR was performed with One-step RT-PCR kit (Qiagen) as previously described [30].

### Whole-mount mRNA *in situ* hybridization

Whole-mount mRNA in situ hybridization was performed as described previously [30]. Dig-labeled antisense probes of *α-catenin* and *chordin* were generated from a 975-nt cDNA fragment encoding N-terminal 325 aa of α-catenin, and entire 570 bp 3′UTR, respectively.

### Western blot analysis

Embryos were deyolked as described previously [31], and dissolved directly by 2×SDS-PAGE loading buffer (2 µl per embryo). The samples were separated on 8% or 12% SDS-PAGE gel (for detection of α-catenin and EGFP protein, respectively). The antibodies against EGFP (Santa Cruz), α-catenin (BD), β-actin (Sigma) and α-tubulin (Sigma) were diluted in 2% BSA as a ratio of 1∶1000, 1∶500, 1∶2000 and 1∶10000, respectively.

### Cell culture and transfection

HEK293T cells were cultured in Dulbecco's modified Eagle's medium (Gibco) supplemented with 10% calf serum in an atmosphere containing 5% CO2. H 1 pol III promoter-driven shRNA and EGFP reporter (pEGFP-C1, Clontech) and DsRed plasmid were cotransfected with a ratio (20∶1∶2) into the HEK293T cells using the calcium phosphate method.

## Supporting Information

Figure S1 Knockdown of *EGFP* gene by H1 pol III promoter shRNA^EGFP-ORF^ in cultured 293T cells. (A) EGFP fluorescence and (B) Western blot analysis of 48 hours post transfection.
(5.36 MB TIF)

Figure S2 Transient knockdown of chromosomally integrated *EGFP* gene. (A) Diagram of mir-shRNA^EGFP-SV40-1^ and mir-shRNA^EGFP-SV40-2^ against the proximal and distal SV40-3′UTR of *EGFP*, respectively. (B) Detection of EGFP and DsRed fluorescence in 24 hpf embryos injected with indicted mRNAs. Red fluorescence was used as an injection control. (C) Western blot analysis of 24 hpf embryos shown in panels B. The β-actin was used as a loading control. (D) Detection of EGFP fluorescence in the Tg(*zgata-1:EGFP-SV40*) transgenic embryos injected with indicated mRNAs. The development and morphology of injected embryos appeared to be normal (bottom panels). (E) Western blot analysis of 24 hpf embryos shown in panels D. The α-tubulin was used as a loading control.
(9.92 MB TIF)

Figure S3 Tissue-specific knockdown of chromosomally integrated *EGFP* transcripts. WISH analysis of *EGFP* and *DsRed* mRNA expression in DsRed^−^ (A), and DsRed^+^ embryos (B) at 30 hpf. MB: midbrain; DN: dorsal neuron; PBI: posterior blood island.
(5.17 MB TIF)

Table S1 (0.06 MB DOC)
