# Supplementary figures and images for: Heritable and Lineage-Specific Gene Knockdown in Zebrafish Embryo
Source: PLoS One. 2009 Jul 3;4(7):e6125. doi: 10.1371/journal.pone.0006125 (PMC2702085; doi:10.1371/journal.pone.0006125)

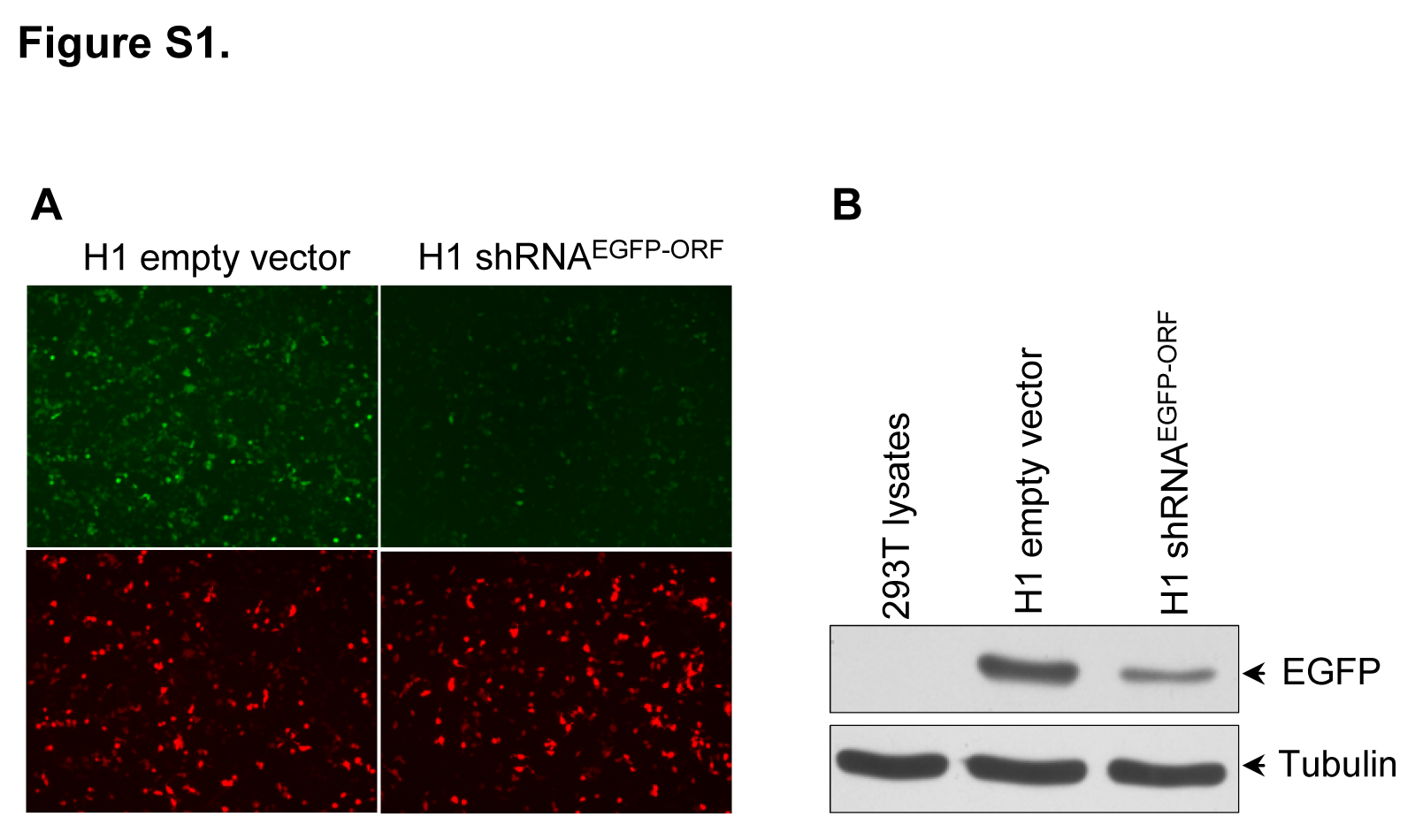

Supplement: Figure S1 — Knockdown of EGFP gene by H1 pol III promoter shRNAEGFP-ORF in cultured 293T cells. (A) EGFP fluorescence and (B) Western blot analysis of 48 hours post transfection. (5.36 MB TIF) [file pone.0006125.s001.tif]

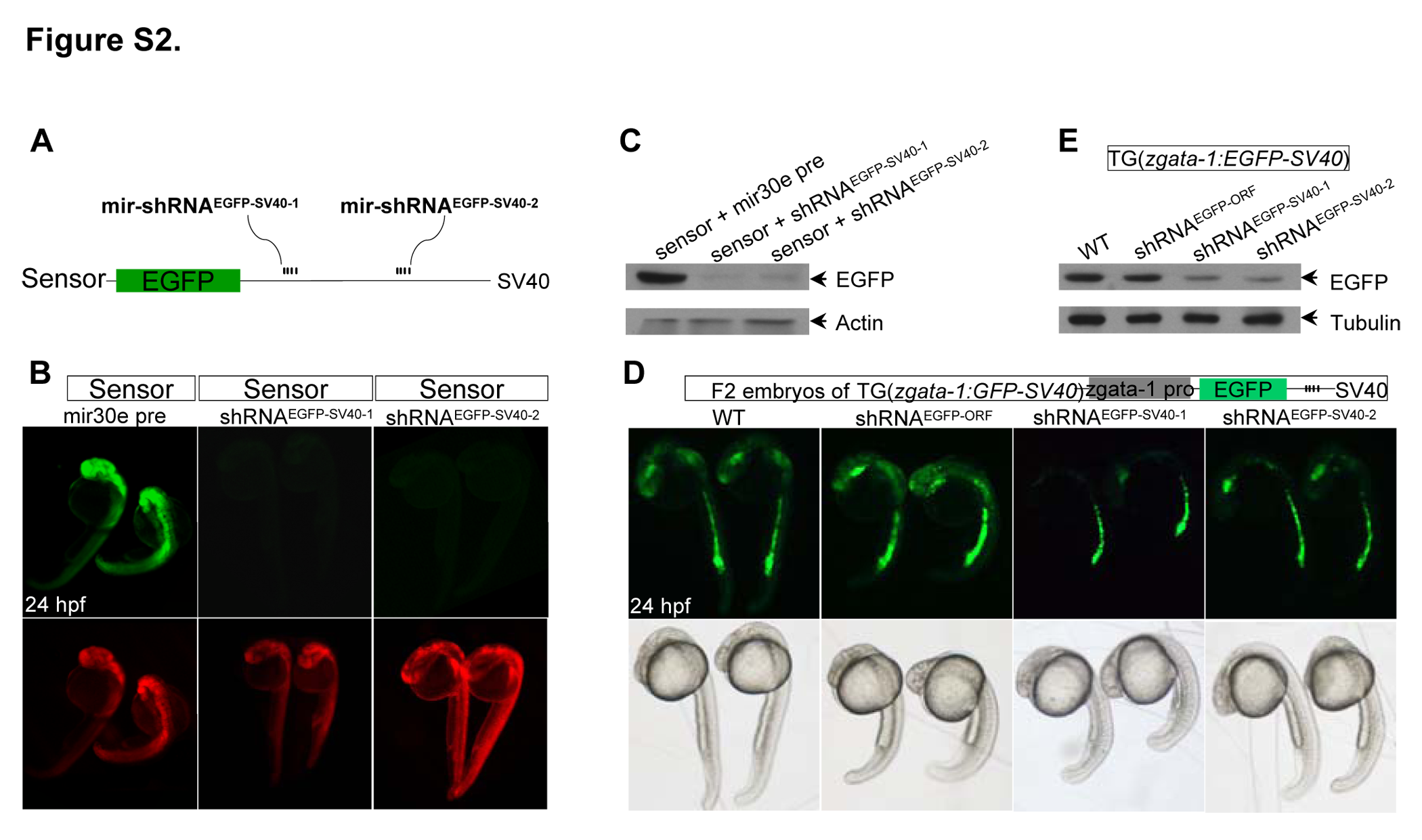

Supplement: Figure S2 — Transient knockdown of chromosomally integrated EGFP gene. (A) Diagram of mir-shRNAEGFP-SV40-1 and mir-shRNAEGFP-SV40-2 against the proximal and distal SV40-3′UTR of EGFP, respectively. (B) Detection of EGFP and DsRed fluorescence in 24 hpf embryos injected with indicted mRNAs. Red fluorescence was used as an injection control. (C) Western blot analysis of 24 hpf embryos shown in panels B. The β-actin was used as a loading control. (D) Detection of EGFP fluorescence in the Tg(zgata-1:EGFP-SV40) transgenic embryos injected with indicated mRNAs. The development and morphology of injected embryos appeared to be normal (bottom panels). (E) Western blot analysis of 24 hpf embryos shown in panels D. The α-tubulin was used as a loading control. (9.92 MB TIF) [file pone.0006125.s002.tif]

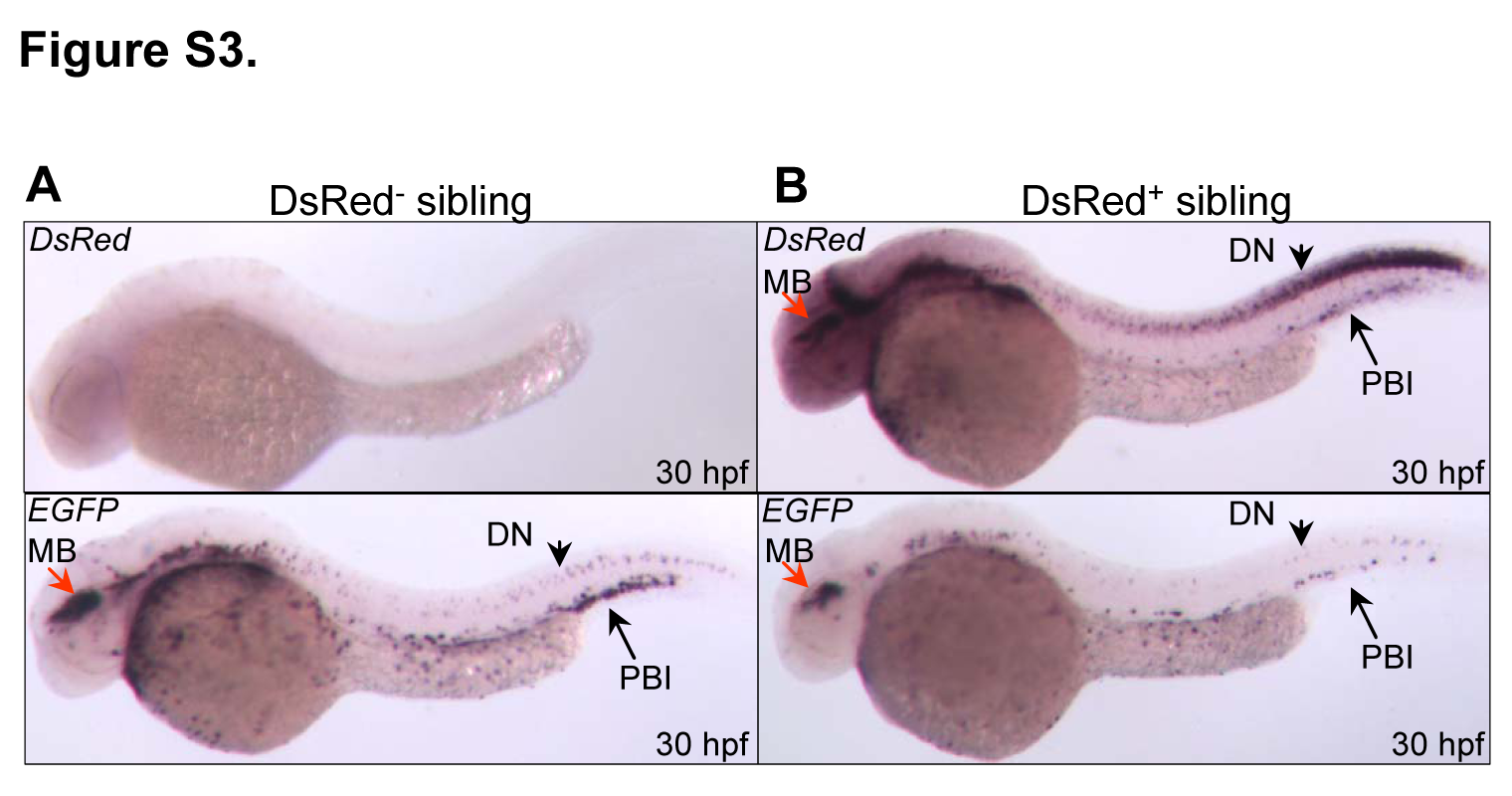

Supplement: Figure S3 — Tissue-specific knockdown of chromosomally integrated EGFP transcripts. WISH analysis of EGFP and DsRed mRNA expression in DsRed− (A), and DsRed+ embryos (B) at 30 hpf. MB: midbrain; DN: dorsal neuron; PBI: posterior blood island. (5.17 MB TIF) [file pone.0006125.s003.tif]
