# Supplementary material for: Heritable and Lineage-Specific Gene Knockdown in Zebrafish Embryo
Source: PLoS One. 2009 Jul 3;4(7):e6125. doi: 10.1371/journal.pone.0006125 (PMC2702085; doi:10.1371/journal.pone.0006125)
Supplement: Table S1 — (0.06 MB DOC) [file pone.0006125.s004.doc]

**Table S1: Primers and oligonucleotides sequences used in this study.**

| **Sequence Name** | **Sequence with restriction site underlined (5’-3’)** | **Description** |
| --- | --- | --- |
| mir30e-F  mir30e-R | ATAGAATTCACAGCCATGCCATAGTTTTAGG  ATCCTCGAGAGTTCATCATATGACCAGTGAC | Cloning of zebrafish mir30e precursor used in **Figure 1.** |
| mir155-F  mir155-R | AACGAATTCTCTGCATTCAGCTTCATACGC  ATTCTCGAGTCAGCTTAACAGTTACATTGG | Cloning of zebrafish mir155 precursor used in **Figure 1.** |
| EGFP-2xPT^mir30e^-F  EGFP-2xPT^mir30e^-R | TCGAGGCTGCAAACATCCGACTGAAAGTAGCTAAGCTGCAAACATCCGACTGAAAGT  CTAGACTTTCAGTCGGATGTTTGCAGCTTAGCTACTTTCAGTCGGATGTTTGCAGCC | Cloning of sensor for detecting mir30e used in **Figure 1.** |
| EGFP-2xPT^mir155^-F  EGFP-2xPT^mir155^-R | TCGAGCCCCTATCACGATTAGCATTAATAGCTAACCCCTATCACGATTAGCATTAAT  CTAGATTAATGCTAATCGTGATAGGGGTTAGCTATTAATGCTAATCGTGATAGGGGC | Cloning of sensor for detecting mir155 used in **Figure 1.** |
| mir30e-linker-F  mir30e-linker-R | ATGTCTTCGTCTAGAAGAAGACAA AGCCAACTGCTGTTACTCTC (Bbs I)  TTGTCTTCTTCTAGACGAAGACAT AGCCCGTACTGCCAGCTG (Bbs I) | Used for replacing mir30e stem-loop region with other shRNA sequences used in **Figure 2A.** |
| β-actin-F  β-actin-R | ATAGGATCCATGGATGAGGAAATCGCTGCCCTG  TCAGAATTCGTCCCATGCCAACCATCACTC | Cloning of zebrafish β-actin genomic fragment, used in **Figure 5.** |
| DsRed-Express-F  DsRed-Express-R | TATGAATTCTCATGGCCTCCTCCGAGGAC  ATACTCGAGCTACAGGAACAGGTGGTGGC | Cloning of DsRed-Express in-frame fusion with β-actin exon 3 used in **Figure 5.** |
|  β-actin-F  DsRed-Express for RT | ATAGGATCCATGGATGAGGAAATCGCTGCCCTG  TTGTAGTCGGGGATGTCGGCG | For detecting splicing by RT-PCR used in **Figure 5.** |
| BGH poly (A)-F  BGH poly (A)-R | ATACTCGAGCGTTTAAACCCGCTGATCA  ATTAGGAATAGCGGCCGCCTTTCCGCCTCAGAAGCCA | Cloning of BGH poly (A) sequence downstream DsRed-Express used in **Figure 5.** |
| mir-shRNA^EGFP-ORF^-F  mir-shRNA^EGFP-ORF^-R | GGCTAGCAGGCTGACCCTTTGAAGTTCACTGGTGCACATGATGGAGTGAACTTCAGGGTCAGCTTGCC  GGCTGGCAAGCTGACCCTGAAGTTCACTCCATCATGTGCACCAGTGAACTTCAAAGGGTCAGCCTGCT | Knockdown in **Figure 2.** |
| EGFP-3’UTR-1xINS-F  EGFP-3’UTR-1xINS-R | TCGAGCGGCAAGCTGACCCTGAAGTTCAT  CTAGATGAACTTCAGGGTCAGCTTGCCGC | Used in **Figure 2C.** |
| EGFP-3’UTR-2xINS-F  EGFP-3’UTR-2xINS-R | TCGAGCGGCAAGCTGACCCTGAAGTTCATAGCTAACGGCAAGCTGACCCTGAAGTTCA  CTAGATGAACTTCAGGGTCAGCTTGCCGTTAGCTATGAACTTCAGGGTCAGCTTGCCGC | Used in **Figure 2C.** |
| mir-shRNA^EGFP-SV40-1^-F  mir-shRNA^EGFP-SV40-1^-R | GGCTAGTAGATCCAGACTTATGATGAGACTGGTGCACATGATGGAGTCTTATCATGTCTGGATCTACG  GGCTCGTAGATCCAGACATGATAAGACTCCATCATGTGCACCAGTCTCATCATAAGTCTGGATCTACT | Knockdown used in **Figure 6.** and **Figure S2.** |
| mir-shRNA^EGFP-SV40-2^-F  mir-shRNA^EGFP-SV40-2^-R | GGCTAACAACTAGAATGTTCAGTGGAAACTGGTGCACATGATGGAGTTTTCACTGCATTCTAGTTGTG  GGCTCACAACTAGAATGCAGTGAAAACTCCATCATGTGCACCAGTTTCCACTGAACATTCTAGTTGTT | Knockdown used in **Figure S2.** |
| mir-shRNA^chordin-3’UTR-1^-F  mir-shRNA^chordin-3’UTR-1^-R | GGCTATGCGCAAACACATTCACAGGAGACTGGTGCACATGATGGAGTCTTCTGTGTGTGTTTGTGCAG  GGCTCTGCACAAACACACACAGAAGACTCCATCATGTGCACCAGTCTCCTGTGAATGTGTTTGCGCAT | Knockdown used in **Figure 3.** |
| mir-shRNA^chordin-3’UTR-2^-F  mir-shRNA^chordin-3’UTR-2^-F | GGCTAGATGCTCATTAGTTCATAAGCTCCTGGTGCACATGATGGAGGAGTTTATGCTAATGAGCATCC  GGCTGGATGCTCATTAGCATAAACTCCTCCATCATGTGCACCAGGAGCTTATGAACTAATGAGCATCT | Knockdown used in **Figure 3.** |
| mir-shRNA^a-catenin-3’UTR-1^-F  mir-shRNA^a-catenin-3’UTR-1^-R | GGCTCGTCGCCAATCTGTTCCTTTCTTTCTGGUGCACAUGAUGGAGAAAGAAAGGCAGATTGGTGACT  GGCTAGTCACCAATCTGCCTTTCTTTCTCCATCATGTGCACCAGAAAGAAAGGAACAGATTGGCGACG | Knockdown used in **Figure 4.** |
| mir-shRNA^a-catenin-3’UTR-2^-F  mir-shRNA^a-catenin-3’UTR-2^-R | GGCTAATGGCTAAACTCTTTTAGGTCTACTGGUGCACAUGAUGGAGTAGACCTAAGAGTTTAGTCATG  GGCTCATGACTAAACTCTTAGGTCTACTCCATCATGTGCACCAGTAGACCTAAAAGAGTTTAGCCATT | Knockdown used in **Figure 4.** |
| H1 pol III-shRNA^EGFP^-F  H1 pol III-shRNA^EGFP^-R | GATCCCGGCAAGCTGACCCTGAAGTTCATTCAAGAGATGAACTTCAGGGTCAGCTTGCCTTTTTGGAAA  AGCTTTTCCAAAAAGGCAAGCTGACCCTGAAGTTCATCTCTTGAATGAACTTCAGGGTCAGCTTGCCGG | Cloning of shRNA^EGFP^ into H1 vector used in **Figure S1.** |
| mir-shRNA^gata1-3’UTR^-F  mir-shRNA^gata1-3’UTR^-R | GGCTAACCATAACGAAGTTCTTGTAAATCTGGTGCACATGATGGAGATTTACAAGCTTCGTTATGGTC  GGCTGACCATAACGAAGCTTGTAAATCTCCATCATGTGCACCAGATTTACAAGAACTTCGTTATGGTT | Knockdown used in **Figure 7.** |
| mir30e-Xho I-F  mir30e-Xba I-Bgl II-R | ATACTCGAGACAGCCATGCCATAGTTTTAGG  AGCTCTAGAAGATCTAGTTCATCATATGACCAGTGAC | Cloning of mir-shRNA duplex used in **Figure 4.** |
| *chordin*-F  *chordin*-R | TGACTCGAGGGAGAAAGAAGAAATGGCAAAGATGGC  TGATCTAGAAACATACAAACTTTATTGTTTAC | Cloning of zebrafish *chordin* 3’UTR in **Figure 3C.** |
| *chordin* morpholino  *chordin* 4-mismatch | ATCCACAGCAGCCCCTCCATCATCC  ATCC**T**CAGCAG**G**CCCT**G**CATC**T**TCC | Used in **Figure 3D.** |
| EGFP probe | GGCAAGCTGACCCTGAAGT | Labeled with dig and used in **Figure 2B.** |
| α-catenin probe | AGTCACCAATCTGCCTTTCTTT | Labeled with dig and used in **Figure 4D, E.** |
